# Effects of Chronic Low-Dose Internal Radiation on Immune-Stimulatory Responses in Mice

**DOI:** 10.3390/ijms22147303

**Published:** 2021-07-07

**Authors:** Abrar Ul Haq Khan, Melinda Blimkie, Doo Seok Yang, Mandy Serran, Tyler Pack, Jin Wu, Ji-Young Kang, Holly Laakso, Seung-Hwan Lee, Yevgeniya Le

**Affiliations:** 1 Department of Biochemistry, Microbiology and Immunology, Faculty of Medicine, University of Ottawa, Ottawa, ON K1H 8M5, Canada; akhan4@uottawa.ca (A.U.H.K.); dyang055@uottawa.ca (D.S.Y.); rkdwldud@catholic.ac.kr (J.-Y.K.); 2 Radiobiology and Health Branch, Canadian Nuclear Laboratories Ltd., Chalk River, ON K0J 1J0, Canada; melinda.blimkie@cnl.ca (M.B.); mandy.serran@cnl.ca (M.S.); tyler.pack@cnl.ca (T.P.); jin.wu@cnl.ca (J.W.); holly.laakso@cnl.ca (H.L.); 3 Centre for Infection, The University of Ottawa, Immunity and Inflammation, Ottawa, ON K1H 8M5, Canada; 4 CANDU Owners Group Inc., Toronto, ON M5G 2K4, Canada

**Keywords:** low dose radiation, natural killer cells, NKG2D, NKG2D ligand

## Abstract

The Linear-No-Threshold (LNT) model predicts a dose-dependent linear increase in cancer risk. This has been supported by biological and epidemiological studies at high-dose exposures. However, at low-doses (LDR ≤ 0.1 Gy), the effects are more elusive and demonstrate a deviation from linearity. In this study, the effects of LDR on the development and progression of mammary cancer in FVB/N-Tg(MMTVneu)202Mul/J mice were investigated. Animals were chronically exposed to total doses of 10, 100, and 2000 mGy via tritiated drinking water, and were assessed at 3.5, 6, and 8 months of age. Results indicated an increased proportion of NK cells in various organs of LDR exposed mice. LDR significantly influenced NK and T cell function and activation, despite diminishing cell proliferation. Notably, the expression of NKG2D receptor on NK cells was dramatically reduced at 3.5 months but was upregulated at later time-points, while the expression of NKG2D ligand followed the opposite trend, with an increase at 3.5 months and a decrease thereafter. No noticeable impact was observed on mammary cancer development, as measured by tumor load. Our results demonstrated that LDR significantly influenced the proportion, proliferation, activation, and function of immune cells. Importantly, to the best of our knowledge, this is the first report demonstrating that LDR modulates the cross-talk between the NKG2D receptor and its ligands.

## 1. Introduction

Humans are continuously exposed to ubiquitous low doses of ionizing radiation (IR) through anthropogenic and natural sources, such as terrestrial, solar, and cosmic radiation. High doses of IR are associated with impaired cellular and physiological functions, significantly reducing organismal lifespan. The “linear-no-threshold” (LNT) model has been used to assess health risks associated with radiation exposure [1]. According to the LNT hypothesis, radiation exposure increases cancer risk in a linear fashion, no matter how low the dose may be [2,3]. However, several lines of experimental evidence suggest that low-dose radiation (LDR) elicits a non-linear response in biological systems and may result in beneficial effects, such as activation of anti-tumor immunity and cellular antioxidant responses to environmental insults [4,5,6]. The detrimental effects of high-dose radiation (HDR) are well known [7,8,9]; however, at low doses, there is a lot of debate as to the actual shape of the curve [10,11,12,13,14,15], warranting further studies.

Generally, radiation with a cumulative dose of up to 100 mGy is referred to as low-dose radiation [16]. Several studies have demonstrated that exposure to low-dose radiation can protect from the damaging effects of subsequent high-dose radiation exposure. This adaptive response has been termed radiation conditioning hormesis [17,18]. One important constituent of the adaptive radiation response is the activation of DNA damage repair pathways [19]. This defense mechanism to oxidative stress, induced by LDR, can provide an endogenous protective response against an injury caused by a subsequent severe stress (e.g., high radiation dose) [18]. The LDR-induced stress can also result in epigenetic reprogramming, activation of various anti-oxidant genes and transcriptional factors, and alterations of several signaling pathways, leading to a prolonged lifespan [20,21,22]. The beneficial effects of low-dose radiation have also been observed in various animal and avian models [4,21,23,24,25,26].

The most imperative risk measure of radiation exposure is cancer development [10,27]. LNT predicts increased cancer risk from doses, derived from epidemiological studies and primarily from high dose radiation (HDR) exposures. However, cancer risk may not always be positively correlated with radiation dose [12,14,28]; depending on the radiation dose rate and level, defensive mechanisms of biological systems are differentially activated by LDR and may suppress cancer induction [12]. Radiation-induced protective pathways are more efficient in the low-dose range [6]. Presumably, these low doses of ionizing radiation (i.e., mild stress) stimulate protective systems that may represent natural anticancer mechanisms [29]. Intriguingly, cancer incidence rates in high natural background radiation areas are lower than those in low background radiation areas [30]. Similarly, workers exposed to various sources of radiation had a lower cancer incidence than expected based on the LNT model [4,31,32,33,34,35,36].

The immune system has a vital role in the defense mechanism against cancer and various environmental insults. Ionizing radiation has been shown to affect the immune system [15,37,38,39], but the impact of LDR on the immune system remains quite ambivalent [40,41,42]. Research has shown that LDR provokes immune-stimulatory responses in primary human monocytes [38], demonstrating that radiation-induced macrophage activation is critical in tumor response [43]. Similarly, LDR exposure triggers selective removal of precancerous and other aberrant cells through intracellular signaling, particularly stimulating anticancer immunity [5]. Low and moderate radiation doses induce transmigration and chemotaxis of activated macrophages [44] and increased phagocytic rate [45]. Moreover, mice exposed to low-dose gamma rays effectively suppress tumor growth by increasing glutathione, thereby enhancing natural killer (NK) cell activity and antibody-dependent cellular cytotoxicity [46]. In addition, repeated irradiations in mice augment the cytotoxicity of NK cells and CD8^+^ T lymphocytes by boosting the IFNγ production of splenocytes [47].

The effects of LDR on the immune system from the perspective of tumorigenesis have been elucidated in very few studies. Investigating the effects of low-dose radiation has always been a significant challenge. The available epidemiological data with external radiation exposures has insufficient statistical significance and often inadequate methodological approaches. Additionally, there are many confounding factors to be considered in the analysis of endpoint phenotypes [12,13,41,48,49]. Importantly, both the total radiation dose and the dose rate are important factors that affect a response in a biological system and are important to distinguish and investigate. Finally, LDR-induced changes in the cellular immune response and the consequent effects on mammary cancer have not been investigated using a mammary tumor model. To address this gap in knowledge, this study utilized an in vivo mouse model to assess the effects of chronic internal low-dose beta radiation on the development and progression of mammary cancer. This study exploits a transgenic mouse model (FVB/N-Tg(MMTVneu)202Mul/J). These mice are characterized by the over-expression of *neu* gene, a rat homolog to the human *her2* gene. As a result, MMTV-Neu mice spontaneously develop mammary adenocarcinomas with a mean tumor latency of 7.5 months. The first tumors appear as early as at 4 months of age. Disease progression involves lung metastasis, with 75–80% of mice developing lung metastases by the age of 7–8 months. The mean time of death for these animals is 11–12 months [50,51,52]. Importantly, in this mouse model, the overexpression of *neu* gene is specific to mammary tissue; thus, no other physiological systems, including the immune system, are affected by this genetic modification, allowing the study of the effects on the native immune system. The radiation facility at Canadian Nuclear Laboratories (CNL) is uniquely designed to accommodate the exposure of experimental animals to both internal and external sources of radiation. Moreover, this state-of-the-art facility enables precise irradiation of animals from low to high doses over a defined period of time. During this study, Tritium (^3^H) was used as a source of radiation due to substantial public concern for potential health effects and the growing international attention [53]. This isotope of hydrogen is a by-product of the nuclear industry and was found to have effects on the biological system during recent investigations at CNL [54,55,56]. To the best of our knowledge, this is the first study that investigates the effects of internal beta LDR on the immune system in an in vivo model of spontaneous tumorigenesis.

## 2. Results

### 2.1. LDR Affects Immune Cell Frequency

**Figure 1:**
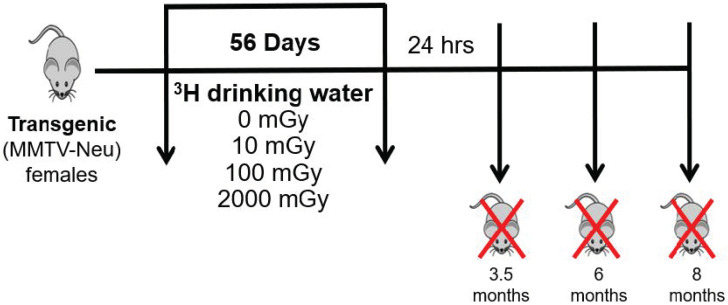
Schematic diagram of the study. Transgenic “MMTV-Neu” female mice underwent chronic low-dose tritium exposure (via drinking water), starting at 1.5 months of age, to a total dose of 0, 10, 100, and 2000 mGy over 56 days. Mice were sacrificed at three time-points: 3.5, 6, and 8 months of age. Blood, spleen, lungs and mammary glands were collected for further processing and analysis. Tumor number and volume were assessed in tumor-bearing mice at the time of sacrifice.

The goal of this study was to investigate the impact of chronic low-dose tritium exposures on the immune status of mice with respect to the development and progression of mammary cancer after internal beta irradiation. To achieve this, MMTV-Neu mice that spontaneously develop mammary adenocarcinomas were employed. These animals received a total dose of 10, 100, or 2000 mGy in drinking water over 56 days. Mice were sacrificed at three time-points to assess cellular response: 3.5, 6, and 8 months of age (Figure 1).

**Figure 2:**
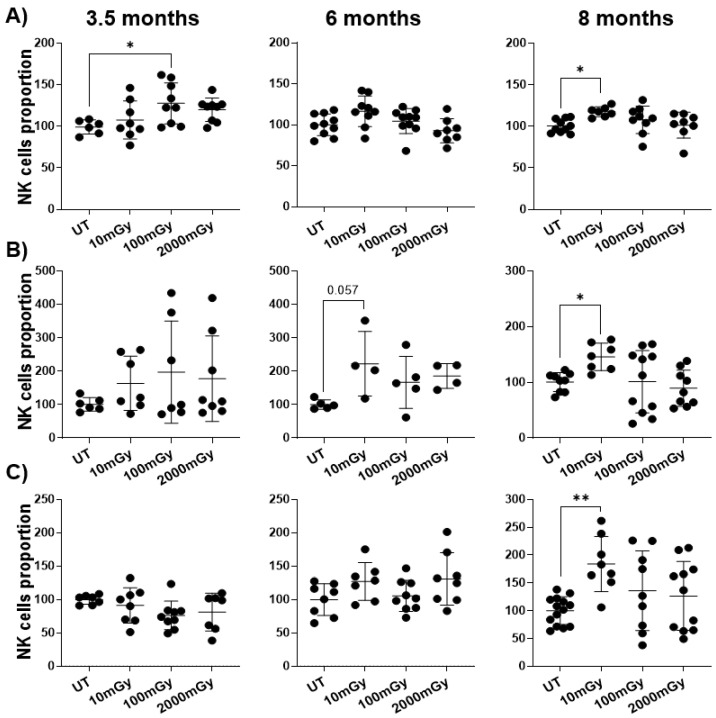
LDR increases NK cell proportion. Single-cell suspensions were generated from different organs of mice sacrificed at indicated time points after radiation exposure. Cells were stained with NK cell surface marker, and relative proportion of NK cells was assessed in (**A**) spleen, (**B**) lungs, and (**C**) mammary glands of untreated control and LDR-exposed mice by flow cytometry. Y-axis represents the relative percentage of NK cells in the total population of mononuclear cells. Percent NK cells in untreated mice were set to 100%. Data represent mean + SD. * *p* < 0.05, ** *p* < 0.01.

The number of various immune cell populations is indicative of the type and strength of the response. Therefore, to investigate the effect of LDR on immune cells, first, the proportion of various immune cell populations was assessed in different tissues (spleen, lungs, and mammary glands). The frequency of NK cells in these tissues was increased for 8-month old mice that received 10 mGy beta internal radiation in comparison to untreated controls (Figure 2). A trend toward increased NK cell proportion was also observed at earlier time points (3.5 and 6 months), with statistical significance noted only in the spleen at 3.5 months. Interestingly, high dose radiation exposure (2 Gy) did not significantly affect the NK cell numbers in the spleen, lungs or mammary glands (Figure 2).

The frequency of T cells (TCRb^+^ NKp46^−^) was evaluated in spleens and lungs. In the spleen, LDR did not significantly affect the frequency of TCRb^+^CD4^+^ T cells at either dose or time-point. However, an increased proportion of TCRb^+^CD8^+^ T cells was observed upon 10 mGy (*p* < 0.198) and 100 mGy (*p* < 0.0253) exposures at 8 months of age, with the 100 mGy increase being statistically significant (Supplementary Figure S1A). Similar to NK cells, high dose radiation exposure did not affect the T cell proportion in the spleen (Supplementary Figure S1A). In lungs, a relationship distinct from that of splenic lymphocytes was noted. TCRb^+^ NKp46^−^ T cells increased at 6 months and decreased at 8 months (Supplementary Figure S1B), whereas TCRb^−^ CD19^+^ B cells demonstrated an opposite trend with decreased frequency at 6 months and increased frequency at 8 months (Supplementary Figure S1C). Trends in both populations were proportional to the radiation dose. An increase in T cells at 6 months and B cells at 8 months was also observed in 2000 mGy irradiated mice (Supplementary Figure S1B,C).

Regulatory T cells (Tregs) play an important role in down-modulating immune responses and are defined, in part, by the expression of the IL-2 receptor-α chain CD25 and the transcriptional factor FoxP3. Changes were not observed in splenic Tregs upon low-dose exposures. However, increases in Tregs were seen in the 2000 mGy groups at 3.5 and 6 months but not 8 months of age (Supplementary Figure S2), suggesting that at high-dose radiation exposures, immune suppressive mechanisms may be activated.

Altogether, these results showed that LDR exposure affected the frequency of the immune cells at different time points. Importantly, the proportion of NK cells was mostly affected by LDR and in all tissues. This suggests that even the low dose of 10 mGy could induce immunomodulation by increasing NK cell proportion, and this effect could still be observed at later time points (Table 1).

### 2.2. LDR Influences Immune Cell Activation Status

The activation status of NK and T cells is suggestive of anti-tumor immunity; immune cells are able to kill target cells via various mechanisms following activation [57]. The activation status of splenic NK and T cells was assessed by flow cytometric analysis of activation-associated surface markers. CD43 is a transmembrane glycoprotein, which is expressed on activated hematopoietic cells. Its signaling can induce chemokine synthesis and cytotoxic activity in NK cells [58]. Similarly, CD69 is a very early marker of lymphocyte activation, which is expressed on T cells after TCR/CD3 engagement [59].

**Figure 3:**
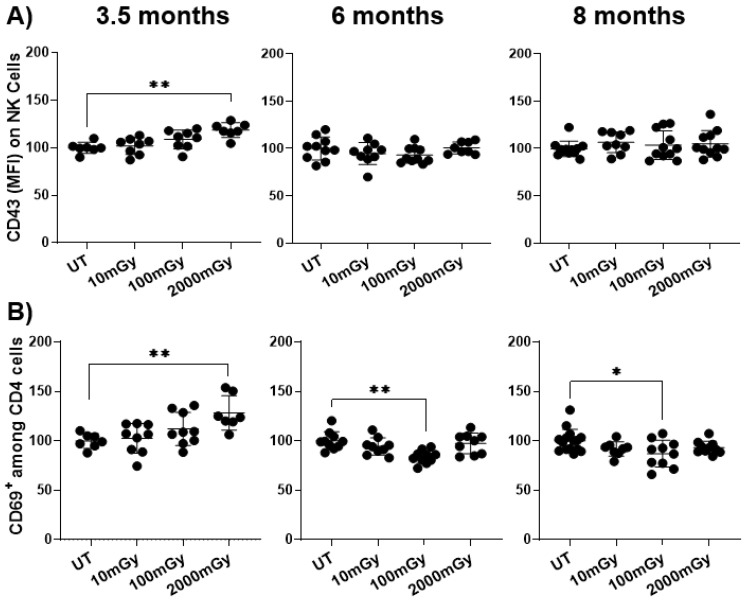
Radiation induces immune cell activation. Splenic lymphocytes from mice sacrificed at indicated points after radiation exposure were used to measure the expression of activation markers. (**A**) Expression of CD43 (activation-associated glycoform) on NK cells and (**B**) expression of CD69 (early activation marker) on CD4+ T were measured by flow cytometry after surface staining. Y-axis in (**A**) represents the relative MFI and (**B**) relative percent of CD4+ cells expressing an activation marker. MFI and percent cells in untreated mice were set to 100%. Data represent mean + SD. * *p* < 0.05, ** *p* < 0.01.

A dose-dependent increase was observed in the expression of CD43 in NK cells and CD69 in CD4^+^ T cells at 3.5 months but not at the later time-points, with the 2000 mGy dose reaching statistical significance (Figure 3A,B), as observed previously [39]. Interestingly, at the later time-points of 6 and 8 months, CD69 expression on CD4^+^ T cells demonstrated a dose-dependent decrease with the 100 mGy dose reaching statistical significance. This suggests a distinct temporal pattern of T cell activation to that of NK cells. Although statistically significant effects were only observed with HDR, these results indicated that radiation exposure can lead to the activation of both NK and T cells, but these effects were transient as they were only observed at an early age, soon after the mice were taken off the tritiated water.

### 2.3. LDR Induces Inflammatory Responses

**Figure 4:**
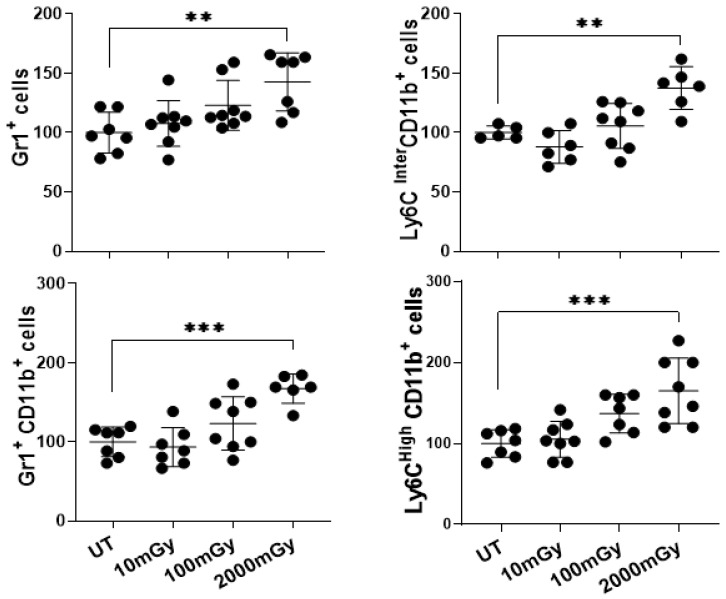
High-dose radiation promotes inflammatory conditions in vivo. Single-cell suspension of splenic lymphocytes was stained with various surface markers to identify, using flow cytometry, the proportion of inflammatory cell populations in mice sacrificed at 3.5 months of age and following LDR exposure. Y-axis represents the percentage of cells relative to the total population of mononuclear cells. Percent cells in untreated mice were set to 100%. Data represent mean + SD. ** *p* < 0.01, *** *p* < 0.001.

It is known that radiation activates a strong pro-inflammatory immune response in the tumor microenvironment, provoking tumor eradication by immune cells [60]. In our study, the contribution of LDR to the activation of inflammation was assessed. Notably, an increasing trend in the proportion of various subpopulations of immune cells involved in inflammation was observed. This was assessed via flow cytometry by staining splenic cells for surface markers that are known to be expressed specifically on the inflammatory compartment of immune cells [61]. The frequency of Gr-1^+^CD11b^+^ cells was measured, as these cells are known to be highly prevalent in the spleens of tumor-bearing mice. Additionally, Ly6C^+^ bearing neutrophils, eosinophils, and both subsets of monocytes/macrophages were assessed in mouse spleens [61,62]. A significant increase in the proportion of inflammatory cells was only observed following high-dose radiation exposure, and only at the early time-point of 3.5 months (Figure 4). At 8 months of age, however, this effect was lost or completely reversed (Supplementary Figure S3).

Altogether, these results suggested that consistent with the previous reports [60,63], high doses of IR induced inflammatory conditions in vivo. However, the effect was not sustained once irradiation was discontinued, as shown by the reduced proportion of inflammatory cells at 8 months. Although statistically significant results were observed only with HDR, a slight trend of positive association of radiation dose and inflammatory cells was noted for the 100 mGy dose as well. Taken together, these results indicated that high dose radiation induced inflammatory conditions in vivo but the effects were acute and subsided with time.

### 2.4. LDR Affects the Function of Immune Cells

Following immune stimulation, the release of cytokines is indicative of immune cell function. Exposure to LDR has been known to enhance cytokine secretion and cytotoxic activity of NK cells [64]. To evaluate the IFNγ production of NK cells upon various stimulations ex vivo, splenic lymphocytes were isolated from mice in each group and were stimulated with either IL-2/IL-12, or anti-NKp46 for 4 h. In order to measure the IFNγ production of T cells, splenic lymphocytes were stimulated with anti-CD3/28 for 16 h followed by intracellular staining.

**Figure 5:**
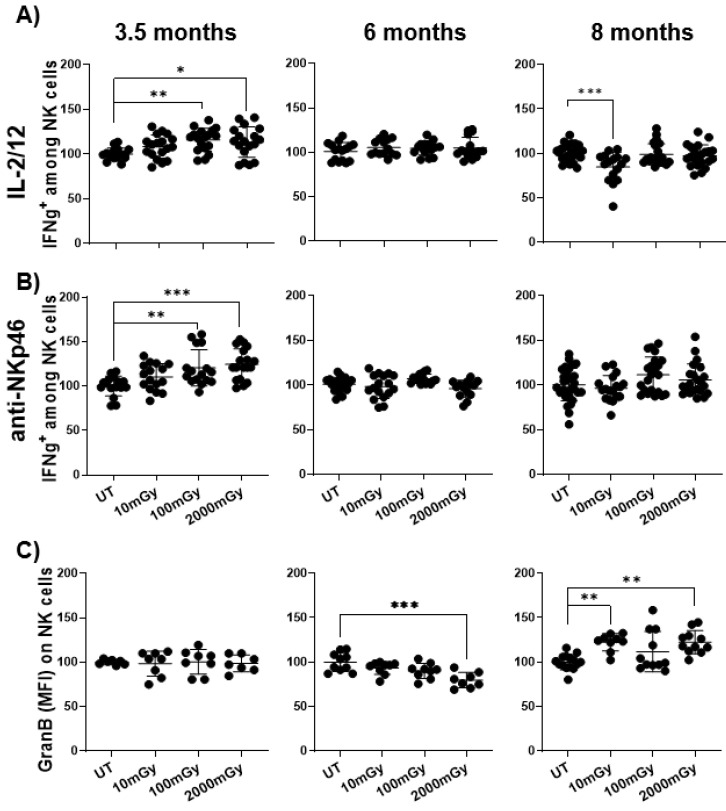
LDR enhances immune cell function. Splenic lymphocytes isolated from mice sacrificed at indicated time points after radiation exposure were stimulated with either (**A**) IL-2 and IL-12 or (**B**) anti-NKp46 for 5 h followed by intracellular staining and flow cytometry to measure IFNγ positive cells in the NK cell population. (**C**) Intracellular staining of splenic lymphocytes was performed to measure expression of Granzyme B in NKp46^+^ DX5^+^ NK cells by flow cytometry. To measure IFNγ, the experiment was performed in triplicate. Y-axis in (**A**) represents the relative percent of IFNγ^+^ NK cells and (**B**) represents the relative MFI of Granzyme B staining on NK cells. Percent cells and MFI in untreated mice were set to 100%. Data represent mean + SD. * *p* < 0.05, ** *p* < 0.01, *** *p* < 0.001.

We observed that LDR exposure increased the proportion of NK cells producing IFNγ upon stimulation with cytokines or activating receptors at 3.5 months. The enhanced response was significant with LDR exposure for the 100 mGy and high-dose control (2000 mGy) groups. However, this effect subsided in the 6 and 8 month time points (Figure 5A,B). On the contrary, differences in the proportion of IFNγ producing T cells were not observed in either treatment group or time-point. 

In conjunction with IFNγ production, cytotoxic cells also mount effective killing mechanisms to target cells via the release of cytolytic enzymes, including Granzyme B. Thus, we investigated whether LDR can enhance Granzyme B production. Although there was no difference in the proportion of granzyme B^+^ NK cells in either treatment group at 3.5 and 6 months, we observed an increase in Granzyme B-producing NK cells in mice receiving a dose of 10 mGy and 2000 mGy at 8 months (Figure 5C). A similar observation was made for T cells, where 10 mGy LDR exposure increased Granzyme B^+^ CD4 and CD8 cells at 8 months (Supplementary Figure S4A,B). Collectively, these results indicated that exposure to LDR can have a significant impact on immune cell functional plasticity by inducing cytokine production of NK cells and cytotoxicity of NK and T cells.

### 2.5. LDR Suppresses Immune Cell Proliferation

**Figure 6:**
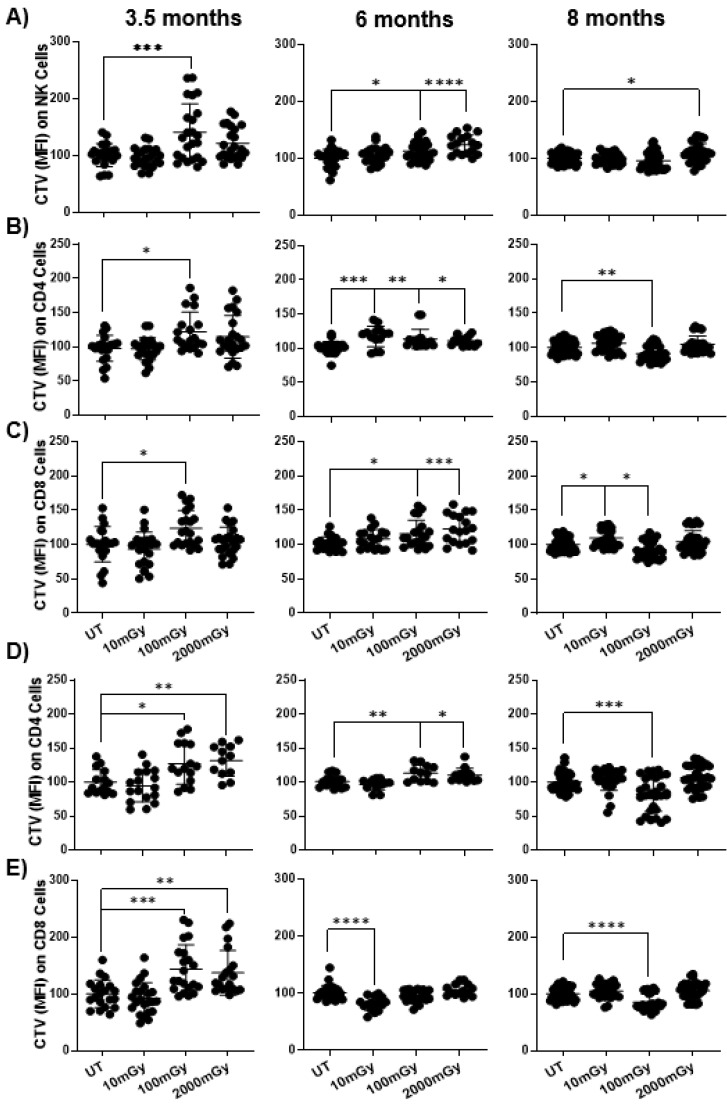
LDR suppresses immune cell proliferation. Single-cell suspensions were obtained from spleens of mice sacrificed at indicated time points after radiation exposure. Cells were stained with cell trace violet (CTV) dye and stimulated with (**A**–**C**) IL-2 (1000 U/mL) or (**D**,**E**) anti-CD3/28 for three days. The MFI of CTV dye in NK, CD4+ and CD8+ cells was measured using flow cytometry. The experiment was performed in triplicate. Note, the higher intensity of the CTV dye indicates lower proliferation as the dye gets diluted upon cell division. Y-axis indicates the relative MFI percentiles among different treatment groups. MFI of untreated mice was set to 100%. Data represent mean + SD. * *p* < 0.05, ** *p* < 0.01, *** *p* < 0.001, **** *p* < 0.0001.

Anti-tumor immunity is highly modulated by the proliferation of immune cells. To determine the proliferative potential of NK and T cells, CellTrace Violet (CTV) dye dilution was measured upon IL-2 or anti-CD3/28 stimulation, followed by 3 days of ex vivo culture. In the assay, the higher intensity of CTV dye indicates a lower proliferation. The results demonstrated a decrease in NK cell proliferation in the 100 mGy group at 3.5 months; in both the 100 and 2000 mGy groups at 6 months; and in 2000 mGy group at 8 months (Figure 6A). Similarly, upon IL-2 stimulation, proliferation of CD4 and CD8 T cells decreased at 3.5 months in the 100 mGy group and at 6 months in the 100 and 2000 mGy groups, but increased at 8 months in the 100 mGy group (Figure 6B,C). A similar pattern was observed when cells were stimulated with anti-CD3/28. There was a reduced proliferation for both CD4 and CD8 T cells at 3.5 months and 6 months for CD4 T cells following 100 mGy and 2000 mGy irradiation. Increased proliferation was observed for CD8 T cells at 6 months in the 10 mGy group, and for both CD4 and CD8 T cells at 8 months with 100 mGy dose (Figure 6D,E). Taken together, these results suggest that LDR exposure is critical for immune cell proliferation and has suppressive effects on NK and T cell proliferation at early time points and stimulating effects on T cells at later stages (Table 2).

### 2.6. LDR Regulates Cross-Talk between NKG2D Receptor and Its Ligand

**Figure 7:**
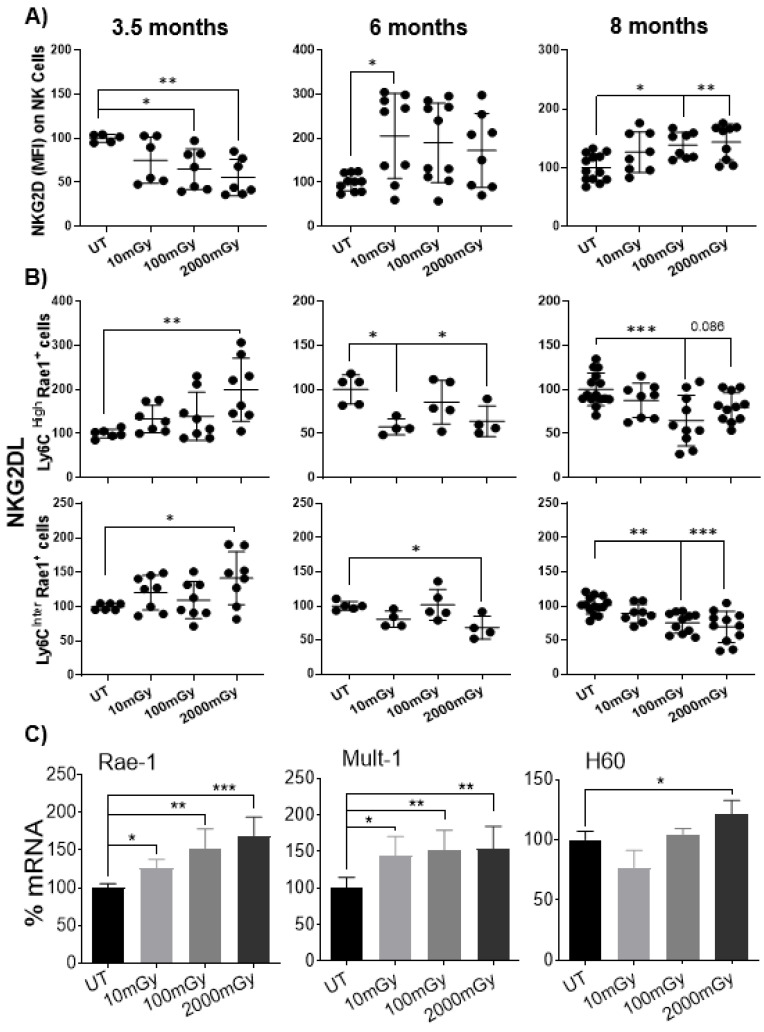
LDR modulates a cross-talk between NKG2D and its ligands. Isolated splenic cells from untreated and irradiated mice were stained with NKG2D or one of its ligands, Pan-Rae-1, prior to flow cytometry analysis. (**A**) Expression of NKG2D (MFI) on NK cells and (**B**) proportion of Ly6C+ cells expressing Pan-Rae1, a family of NKG2D ligands (NKG2D-L), on splenic leukocytes were measured. Y-axis in (**A**) represents the relative MFI of NKG2D staining and (**B**) relative percent of Ly6C+ cells expressing NKG2D-L. MFI and percent cells in untreated mice were set to 100%. (**C**) mRNA expression levels of NKG2DL (Rae-1, Mult-1 and H60) in spleens of mice from 3.5-month time point. Data represent mean + SD. * *p* < 0.05, ** *p* < 0.01, *** *p* < 0.001.

The most noticeable observation revealed in this study was the reduced expression of natural killer group 2, member D (NKG2D) receptor on splenic NK cells of mice exposed to LDR (Figure 7A). NKG2D is one of the activation receptors expressed on NK cells that binds to a diverse family of ligands and plays a critical role in NK cell-mediated immune response to transformed cells [65,66,67,68]. A variety of NKG2D ligands (NKG2DL) on the cell surface can become upregulated during stress conditions produced by diverse stimuli, including radiation, although their expression is regulated at multiple levels [68,69,70,71,72]. To characterize anti-tumor surveillance of NK cells, the expression of NKG2D on the splenic NK cells of mice exposed to LDR was measured. Notably, NKG2D expression was found to be reduced on NK cells at 3.5 months in a radiation-dose-dependent manner (Figure 7A). Reduced expression of NKG2D on NK cells may suggest increased expression of NKG2DL on target cells, using a feedback regulation in which sustained expression of NKG2DL on target cells can lead to the internalization of NKG2D on NK cells [73]. Consistent with this, we observed increased expression of Rae-1 (NKG2D ligand) on activated splenic leukocytes (defined by the Ly6C expression) of mice that received the LDR exposure (Figure 7B). Surprisingly, the effect of decreased NKG2D expression was not sustained and the opposite trend was observed with increased NKG2D expression on splenic NK cells and a coincident decrease of pan-Rae-1 on activated splenic leukocytes at 8 months (Figure 7A,B).

To investigate further the correlation between the NKG2D downregulation on NK cells and NKG2D-L upregulation on other stressed cells, the expression of individual members of the NKG2D-L family (Rae-1, Mult-1 and H60) was assessed at the transcriptional level for the 3.5-month time point. Interestingly, HDR upregulated the expression of all stress ligands, however, with LDR only the expression of Rae-1 and Mult-1, was upregulated compared to control mice (Figure 7C), suggesting an LDR-specific response and, perhaps, pointing toward a potential LDR biomarker/sensor. These findings support our hypothesis that low-dose radiation activates NKG2D-related pathways and modulates the cross-talk between NKG2D and its ligands.

### 2.7. LDR Has No Impact on Tumorigenesis at Organismal Level

**Figure 8:**
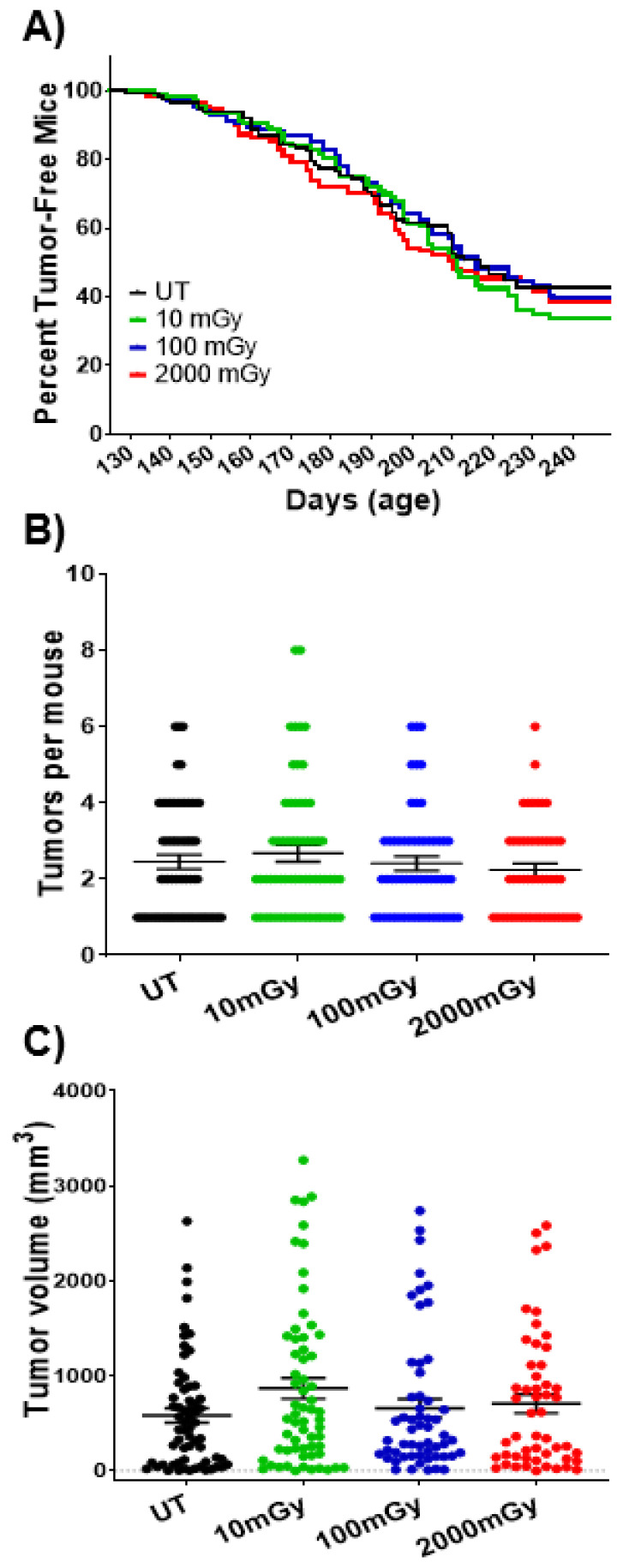
LDR has no impact on tumor development and burden. Transgenic “MMTV-Neu” female mice were exposed to total doses of 0, 10, 100, and 2000 mGy of chronic low-dose tritium. Exposures started at 1.5 months of age and were delivered via drinking water over 56 days. These transgenic mice develop their first spontaneous tumors at the age of 4 months. (**A**) Percentage of tumor-free mice was measured starting from the age of 130 days. (**B**) Number of tumors per mouse and (**C**) tumor volume were measured in tumor-bearing mice at the time of sacrifice. Data represent mean + SD.

Finally, in order to explore the effects of LDR on the immune system and how those further affect the process of tumorigenesis at the organismal level, the number and size of tumors from individual mice (*n* = 550) were measured upon sacrifice. There was no statistical difference among different treatment groups (Figure 8A). Similarly, no significant differences in the tumor size or number were observed between the control mouse group and that exposed to various doses of radiation (Figure 8B,C). These data suggest that although immune changes were detected at the cellular and molecular levels, the LDR effects were not propagated to the organismal level. This may be due to the transiency of the effects on NK cell immunomodulation that were primarily observed at early time points, soon after the irradiation was stopped. This could also be due to other systemic compensatory mechanisms stemming from the nature of the transgenic murine strain.

Interestingly, the high-dose control group (2000 mGy) did not exhibit any acceleration in tumorigenesis or increased tumor load, as would be expected based on the LNT model. This points to the importance of the dose rate, as well as the total dose received, in the observable tissue and systemic effects. In this study, low dose rates of exposure were used for 2000 mGy total cohort—910 MBq/L. This finding supports the notion that the detriment of high-dose radiation exposure can be diminished, and perhaps even circumvented via a decreased dose rate, which can be achieved through chronic or fractionated regimens. The latter is being employed in radiotherapy, e.g., total body irradiation for leukemic patients.

## 3. Discussion

It would be difficult to imagine life without radiation, especially in modern medical care where every medium-sized hospital is equipped with crucial, radiation-emitting equipment. Moreover, about 10% of the world’s electricity is estimated to be generated by nuclear power plants [6]. Generally, the radiation protection framework employs the “linear no-threshold” (LNT) model that was originally adopted at the end of the 1950s. According to the LNT theory, any dose of ionizing radiation, no matter how low it is, increases cancer risk, and the only safe radiation level is “ZERO”. This model is the basis for the existing radiation protection regulations. However, several studies have called into question the LNT model of radiation protection, since radiation-related carcinogenicity at low and intermediate doses is highly stochastic. Neither the large-scale nuclear incidents nor the atomic bombings of Japan provided statistically significant epidemiological evidence to support the LNT model at the low-dose/dose rate range (≤100 mGy; 6 mGy/h) [6,12,74,75,76,77]. It is well accepted that high dose/quantity radiation is detrimental. However, experimental and epidemiological studies have shown that low doses of ionizing radiation can have substantial beneficial effects in biological systems [78,79]. Currently, the field of low-dose effects is the most controversial and highly discussed topic in radiation biology.

Prolonged and acute exposure to LDR can have a significant impact on immune cell proportion [37,41]. As an integral part of the innate immune system, NK cells play a crucial role in immune surveillance. In our study, an increased NK cell frequency was observed in various organs in mice that received LDR exposure. Others have shown that LDR can enhance the activation and function of immune cells (particularly NK cells) with regard to IFNγ and Granzyme B production [25,39]. In our study, these effects were transient, and enhanced IFNγ production was not observed at later time points, i.e., several months after radiation exposure. On the other hand, an augmented Granzyme B production was noted in NK and T cells from irradiated mice at the latest time point (8 months).

In agreement with our results, it has been shown that radiation can increase NK cell proportion, activation, and killing potential [37,46,47]. Radiation therapy was shown to increase NK cell cytotoxicity [80]. Similarly, IL-2 primed purified NK cells showed augmented cytotoxicity upon moderate irradiation [81]. The possibility for LDR-induced direct stimulation of NK cells, via the P38-MAPK pathway, also strengthens our findings [64] that LDR can potentially enhance NK cell’s functional potential.

Inflammation is part of the immune defense mechanisms and is an important contributor to tumor suppression. We observed a significant increase in the proportion of various immune cell subsets involved in the inflammation processes in mice that received HDR. This was consistent with previous findings indicating the role of HDR in inflammation [63]. A slight, non-significant increase, in 3 out of 4 measured subsets, was also observed for the 100 mGy group. However, even with HDR, as the time following irradiation increased, the effects subsided. Similar to HDR mechanisms, higher doses in the LDR range may activate the interconnected network of cytokines, cellular signaling pathways, adhesion molecules, and reactive oxygen species (ROS), which collectively promote a pro-inflammatory microenvironment. LDR was shown to functionally regulate a variety of inflammatory processes and pathways [60,82]. Irradiation has been observed to provoke the activation of inflammasomes in several types of cells, including NK and T cells [83]. Moreover, the observation that tumor-associated macrophages in irradiated tissue have enhanced secretion of pro-inflammatory cytokines IL-6, IL-12, TNF-α, and IFNγ further supports our results [84,85,86].

Our study demonstrated that LDR could suppress NK and T cell proliferation following IL-2 stimulation at 3.5 and 6 month time points. Interestingly, while suppression of T cell proliferation was observed during early time points (particularly at 3.5 months i.e., right after irradiation), the proliferation of CD8^+^ T cells increased at later stages following stimulation. Several studies have observed disparity in cellular proliferation competence after radiation exposure. LDR has been shown to enhance cell proliferation in a cell type-dependent manner through the activation of cellular signaling pathways [87]; however, it has also been observed that IR can induce DNA damage leading to cell cycle arrest, senescence, or apoptosis [88,89,90]. Moreover, IR can cause cell division failure and loss or abnormal distribution of chromosomal material during division, thus impacting cellular integrity [91]. Such discrepancies in the literature could be due to the variation in radiation dose rates, radiation types, and cell types. Interestingly, although in our study NK cells had a reduced proliferative capacity, there was an increased frequency of NK cells in different tissues of mice that received LDR exposure. This suggests two possible complementary mechanisms at play, increased mobilization of NK cells to target tissues, and increased maturity and longevity of NK cells, whcih decrease their rate of proliferation. LDR is known to induce antioxidant mechanisms and longevity [20,21,22]; therefore, it is plausible that LDR could increase the lifespan of immune cells by slowing down apoptosis. The LDR-induced suppression of apoptosis in NK cells has already been demonstrated [37]. In fact, exposure to low radiation doses may induce trivial stress factors to enhance protective antioxidant pathways that may contribute to longevity [5,18]. It has recently been determined that background radiation within the natural range of low-dose radiation increases the life expectancy by approximately 2.5 years [92]. In support of this, others have shown that LDR is involved in DNA double-stranded-break repair mechanisms [33,93], which can ultimately delay cell death mechanisms.

This study resulted in a key significant finding that was not reported previously. An LDR-induced cross-talk between NKG2D and its ligands was identified. NKG2D is one of the critical activating receptors of NK cells that recognizes diverse ligands in humans and mice. The ligation of NKG2D with the corresponding ligand is sufficient to activate cytolysis and cytokine production by NK cells [68,72]. The expression of NKG2D was found to be reduced on NK cells of LDR-treated mice, while ligand expression was upregulated on splenic leukocytes. Several studies have shown that various types of stimuli, including radiation exposure, can upregulate a variety of NKG2D ligands on the surface of stressed cells [65,70,72,94], and the expression of these stress molecules can consequently regulate the NKG2D expression levels on NK cells [95]. Moreover, sustained NKG2D ligand expression, even on normal cells, may cause systemic immunosuppression of NK activities and increase tumor susceptibility independent of tumor context [73,96]. The decrease in NKG2D expression is the consequence of two possible mechanisms: (a) NK cells are functionally active and engaged in clearing target cells expressing the corresponding ligand; or (b) NK cells become exhausted due to prolonged encounters with stressed cell-bound ligands. Our observations support the former mechanism (a) for the following reasons. First of all, NK cell dysfunction and exhaustion are associated with profound proliferation [97], but this was not the case in our study as reduced NK cell proliferation was observed. Secondly, exhausted NK cells possess a reduced ability to produce IFNγ upon stimulation; however, we observed increased IFNγ^+^ NK cells in the spleen of mice that received LDR. Thirdly, the downregulation of activation markers is another sign of NK cell exhaustion [98], which was not observed throughout our experiments. In our study, the downregulation of NKG2D was correlated with increased levels of stress ligand expression after LDR exposure. Thus, reduced NKG2D expression on NK cells is most likely due to the consistent engagement with NKG2D-L-bearing stressed cells due to continuous radiation exposure.

As shown in Figure 2, Figure 3, Figure 4, Figure 5, Figure 6 and Figure 7, the majority of observable effects demonstrated some dose-dependent behavior; however, most of the dependencies were not linear. Both threshold and one-dose trends were noted. Based on this, our study did not provide sufficient data to comment on the validity of the LNT model. The authors would also like to highlight that the analysis of data with a view to support or invalidate the LNT theory was outside the scope of this study.

Lastly, it was observed that LDR exposure did not influence overall tumor burden and volume, even though several studies have proposed a positive correlation between LDR exposure and tumor suppression [4,99,100]. This divergence in results could be due to the difference in radiation quality, quantity and dose rate, as well as the study model. In our study, the following critical factors could contribute to divergence: (a) use of a transgenic model characterized by over-expression of an oncogene that allows spontaneous development of mammary tumors and progression to aggressive lung metastasis, i.e., cancer initiation stage is already activated; (b) prolonged continuous chronic irradiation; (c) discontinuation of irradiation before tumor development and cancer progression stages. Moreover, beta internal radiation exposure (i.e., drinking water in our study) would have different biological impacts compared to external radiation exposure (i.e., gamma rays) [53]. A role of myeloid-derived suppressor cells (MDSCs) cannot be ruled out either. An increase in MDSCs was observed with radiation and is known to suppress the immune system [62], rendering tumor development. Finally, an observed decrease in T cell proliferation may have impacted the tumor burden.

Although we did not observe any effects of LDR at the organismal level, LDR was shown to have potent effects on the proportion, activation, and function of immune cells. Ultimately, our study demonstrated that LDR exposure results in increased expression of NKG2D stress ligands on target cells, which mobilizes and modulates NK cell function in the context of NKG2D-NKG2D-L cross-talk. Our data open exciting new avenues for exploring the potential of low-dose-mediated modulation of NK-driven immune surveillance.

## 4. Methodology

### 4.1. Mice

A total of 550 female FVB/N-Tg(MMTVneu)202Mul/J mice (Stock # 002376, Jackson Laboratories) entered the study at 6 weeks of age. Mice were housed in the Specific Pathogen-Free Biological Research Facility at CNL. Six mice were housed per cage in individually ventilated Thoren weaning cages with ad libitum access to food (Charles River Rodent Chow 5075) and reverse osmosis (RO) water. Mice were exposed to chronic low-dose Tritium in drinking water for total doses of 0 (UT), 10, 100, or 2000 mGy over 56 days. The 2000 mGy was used as a high dose control. Before and after tritium exposure, mice received un-irradiated RO water. All animal husbandry and experimental procedures were approved by the Canadian Nuclear Laboratories Animal Care Committee (BRF 15-01), in accordance with the standards of the Canadian Council on Animal Care (CCAC).

The experimental endpoints were 3.5, 6, and 8 months of age. These endpoints were chosen to examine tissues at multiple stages of mammary tumor development; before tumor development (3.5 months); during tumor development (6 months); and high tumor burden/metastasis development (8 months), as shown in Figure 1. Mice were euthanized via exsanguination under Isoflurane gas anesthesia, followed by cervical dislocation. Blood, mammary glands, lung, spleen, and tumor samples were collected from 115 mice for further processing and analysis.

Tumors were measured weekly using calipers. Mice with cumulative tumor volumes greater than 2.9 cm^3^ were euthanized at any point in the study, as per the clinical endpoints determined by the CNL Animal Care Committee.

### 4.2. Tritium Exposure

Biological grade tritium (HTO) was purchased from American Radiolabeled Chemicals, Inc. (ART0194). High concentration HTO was diluted in reverse osmosis (RO) water to create the mouse drinking water concentrations of 4.5, 45.5, and 910 MBq/L, providing the respective total doses of 10, 100, and 2000 mGy over the 56 day ingestion period. The tritium concentrations for mouse drinking water were calculated based on previous research at CNL, which measured the tritium retention in mouse tissues during chronic tritium exposure [56]. Tritium measurements were performed using a Liquid Scintillation Counter (Tricarb 210TR, Perkin Elmer, Waltham, MA, USA) and Ultima Gold scintillation fluid (Perkin Elmer, Waltham, MA, USA).

### 4.3. Cell Isolation

Spleens were harvested upon the sacrifice, and a single-cell suspension was generated using the following process: dissociating tissues using a 3 mL syringe plunger and passing through a 70-μm filter (Bio Basic Canada Inc., Markham, ON, Canada), centrifugation at 1200× *g* rpm for 10 min at 4 °C; and finally, washing with RPMI containing 2% fetal bovine serum (FBS) (Gibco™, Burlington, ON, Canada). The cell pellet was reconstituted in 1 mL of red blood cell lysis buffer (Roche™, Sigma-Aldrich, Oakville, ON, Canada) followed by an immediate vortex. Cells were washed with RPMI containing 2% FBS and filtered using a nylon mesh before counting. To obtain leukocytes from lungs, tumors, and mammary glands, these tissues were dissociated into small pieces using dissection scissors and then mixed with extraction buffer (RP-10) containing 25 U/mL collagenase VIII (Sigma, Mississauga, ON, Canada). Sample homogenization was done at 37 °C using gentle MACS™ Dissociator (Milteney biotech, Gaithersburg, USA) followed by straining with a 70-μm filter. Lymphocyte isolation was performed using a Percoll gradient centrifugation (Percoll^®^, Millipore Sigma, Oakville, ON, Canada) according to the manufacturer’s instructions. Cells were filtered through a nylon mesh before counting. All the assays were performed on splenic lymphocytes; other tissues were used only for immune profiling.

### 4.4. In Vitro Proliferation and Functional Assay

For the proliferation of NK cells, the single-cell suspension of splenic cells was labeled with cell trace violet (CTV) dye using CellTrace™ Violet Cell Proliferation Kit (Invitrogen™, Burlington, ON, Canada) according to the manufacturer protocol. Cells were washed twice with RP-10, followed by 3 days of culture in RP-10 media (RPMI-1640 medium (HyClone™, Fisher Scientific, Saint-Laurent, QC, Canada) containing 10% FBS, 1× penicillin/streptomycin (HyClone™), 2 mM L-glutamine (Wisent Bioproducts, Saint Bruno, QC, Canada), 10 mmol HEPES (Lonza™, Walkersville, MD, USA), 50 μmol 2-mercaptoethanol (Gibco™) in the presence of 1000 U/mL of recombinant human IL-2 (obtained from NCI Preclinical Repository, Frederick, MD, USA). For T cell proliferation, splenic cells were stimulated with anti-CD3/28 following CTV labeling before culturing. For intracellular IFNγ measurements ex vivo, freshly derived spleen leukocytes were stimulated with either a combination of IL-2 (100 U/mL) and IL-12 (10 ng/mL) (eBioscience™, Burlington, ON, Canada), or with anti-NKp46 (BioLegend™, San Diego, CA, USA) for 1 h and then incubated in RP-10 media containing 5 μg/mL brefeldin A (Invitrogen™, Burlington, ON, Canada) for 4 h, followed by intracellular staining.

### 4.5. Antibodies and Flow Cytometry

Single-cell suspensions (1 × 10^6^ cells) were incubated at 4 °C for 10 min with α-CD16/32 (clone 2.4G2, from BioExpress, Kaysville, UT, USA) to reduce non-specific binding. Cells were labeled with various combinations of fluorochrome-conjugated monoclonal antibodies (mAbs) and incubated at 4 °C for 25 min. The following mAbs were used: anti-TCRβ (H57-597), anti-CD8 (53–6.7), anti-CD49b (DX5), anti-IFNγ, anti-CD11b (M1/70), anti-NKG2D (CX5) from eBioscience™; anti-CD19 (1D3), anti-CD4 (RM4-5), anti-F4/80 (T45-2342), anti-NK1.1 (PK136), anti-Ki-67 (B56), anti-CD69 (H1-2F3), anti-Ly6C (AL-21), anti-Gr1 from BD Biosciences™, anti- Rae- Pan specific from Milteney biotech, anti-CD43 (1B11) activation- Glycoform from BioLegend™ and Live/Dead Fixable Yellow Dead Cell Stain from Invitrogen™. The intracellular staining of IFNγ and Granzyme B was performed using Cytofix/Cytoperm protocols (BD Biosciences™). Intracellular staining of Foxp3 was carried out using a Foxp3 staining kit (eBioscience™) according to the manufacturer’s protocol. Cells were acquired using BD LSRFortessa or Thermofisher Attune NxT flow cytometers, and data was analyzed using Kaluza 1.3 Analysis software (Beckman Coulter) or FlowJo (V10).

### 4.6. RNA Extraction and Quantitative Real-Time PCR

Purification of total RNA from the spleens was performed using miRNeasy Mini Kit (Qiagen^TM^) according to the manufactured protocol. Total RNA (1 μg) was subjected to reverse transcription using RT2 First Strand Kit (Qiagen^TM^). Quantitative real-time PCR (qPCR) was conducted using the appropriate primers and a Bio-Rad CFX96 system with SYBR green to determine the mRNA expression levels of genes of interest. Expression levels were normalized to β actin level.

### 4.7. Statistical Analysis

The statistical analysis was performed using One-way ANOVA with post-hoc Tukey HSD test (* *p* < 0.05, ** *p* < 0.01, *** *p* < 0.001, **** *p* < 0.0001) using Prism Version 8 (GraphPad Software, San Diego, CA, USA).

## 5. Conclusions

Our data demonstrated that LDR results in multiple significant changes in molecular and cellular immune parameters that are indicative of immune activation. However, these changes did not translate to an organismal level and low-dose chronic tritium exposure did not affect the overall tumor burden of MMTV-Neu-exposed mice. This study uncovered evidence that LDR affects the cross-talk between NKG2D and its ligands, the first report of its kind, which warrants further investigation. Our findings aim to contribute to a better understanding of low-dose radiation effects on immune processes and hence, the health risks associated with such exposures in an effort to better advise current radiation protection policies and standards.

## 6. Limitations

Further studies are necessary in order to elucidate the molecular mechanisms causing NKG2D downregulation and its role in modulating NK and T cell proportion, cytokine production, and proliferation.

## Figures and Tables

**Table 1:** Summary of LDR-induced NK cell proportion in different tissues.

Tissue	Dose/Time-Point	3.5 mo	6 mo	8 mo
Spleens	10 mGy			
	100 mGy			
	2000 mGy			
Lungs	10 mGy			
	100 mGy			
	2000 mGy			
Mammary Glands	10 mGy			
	100 mGy			
	2000 mGy			

Legend: 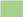 Non-statistical increase, *p* > 0.05, 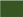 Statistical increase, *p* < 0.05.

**Table 2:** Summary of LDR-induced immune cell proliferation.

Cell Type	Dose/Time-Point	3.5 mo	6 mo	8 mo
NK cells (IL-2 stimulation)	10 mGy			
	100 mGy			
	2000 mGy			
CD4 cells (IL-2 stimulation)	10 mGy			
	100 mGy			
	2000 mGy			
CD8 cells (IL-2 stimulation)	10 mGy			
	100 mGy			
	2000 mGy			
CD4 cells (CD3/CD28 stimulation)	10 mGy			
	100 mGy			
	2000 mGy			
CD8 cells (CD3/CD28 stimulation)	10 mGy			
	100 mGy			
	2000 mGy			

Legend: 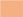 Non-statistical decrease, *p* > 0.05, 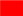 Statistical decrease, *p* < 0.05, 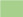 Non-statistical increase, *p* > 0.05, 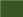 Statistical increase, *p* < 0.05.

## Data Availability

Not applicable.

## Supplementary Materials

The following are available online at https://www.mdpi.com/article/10.3390/ijms22147303/s1.
