# Supplementary material for: Effects of Chronic Low-Dose Internal Radiation on Immune-Stimulatory Responses in Mice
Source: Int J Mol Sci. 2021 Jul 7;22(14):7303. doi: 10.3390/ijms22147303 (PMC8306076; doi:10.3390/ijms22147303)
Supplement: Supplementary file 1 [file ijms-22-07303-s001.zip › ijms-1271721-supplementary.pdf]

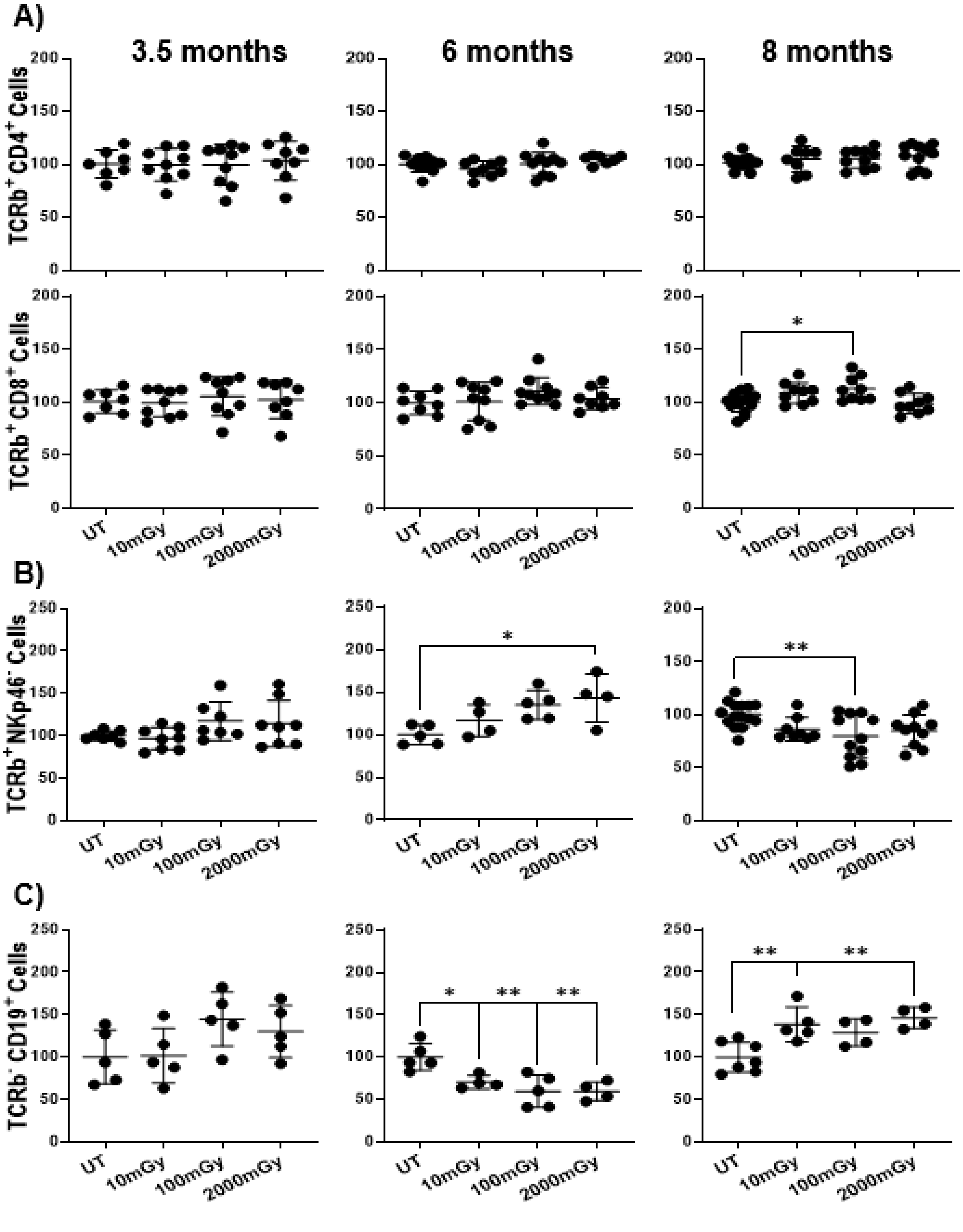

Supplementary Figure 1. Effect of LDR on immune cell proportion.

Mononuclear cells were generated from the spleens and lungs of mice sacrificed at indicated times of age after radiation exposure. Surface staining of cells was performed to analyze the relative proportion of A) T cells (CD4+ and CD8+) in spleen and B) T cells in lungs and C) B cells in lungs of mice received indicated LDR exposure. (Y-axis indicates the relative percentiles of cell proportion among different treatment groups. Cell proportion in untreated mice was reflected as 100%). Data represent mean + SD. * $p < 0.05$, ** $p < 0.01$.

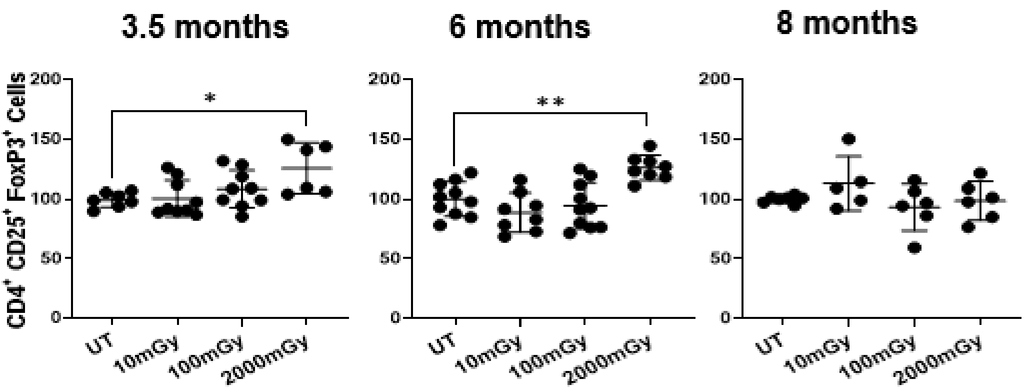

Supplementary Figure 2. LDR increases Treg cell proportion.

Isolated splenic cells from untreated mice or mice sacrificed at the indicated time of age after being exposed to chronic LDR were stained to assess the relative proportion of the regulatory T cells population (CD4+CD25+FoxP3+ cells). (Y-axis indicates the relative percentiles of cell proportion among different treatment groups. Cell proportions in untreated mice were reflected as 100%). Data represent mean + SD. * $p < 0.05$, ** $p < 0.01$.

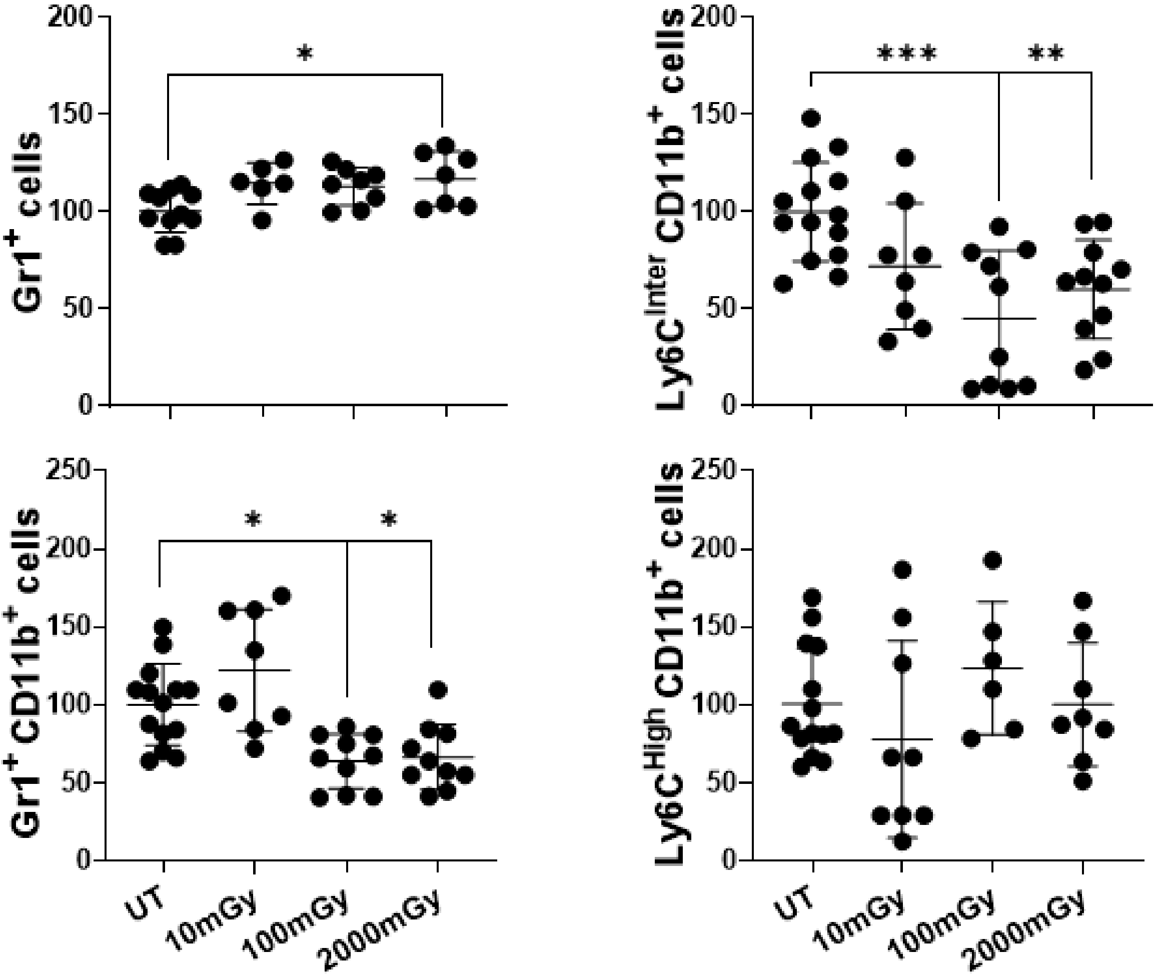

Supplementary Figure 3. Effect of LDR on inflammatory cell proportion.
Single-cell suspension of splenic lymphocytes was stained with various surface markers to identify the proportion of inflammatory cell populations in mice sacrificed at 8 of age after LDR exposed by flow cytometer. Y-axis represents the relative percentage of cells in the total population of mononuclear cells. Percent cells in untreated mice were set to 100%. Data represent mean + SD. * $p < 0.05$, ** $p < 0.01$, *** $p < 0.001$.

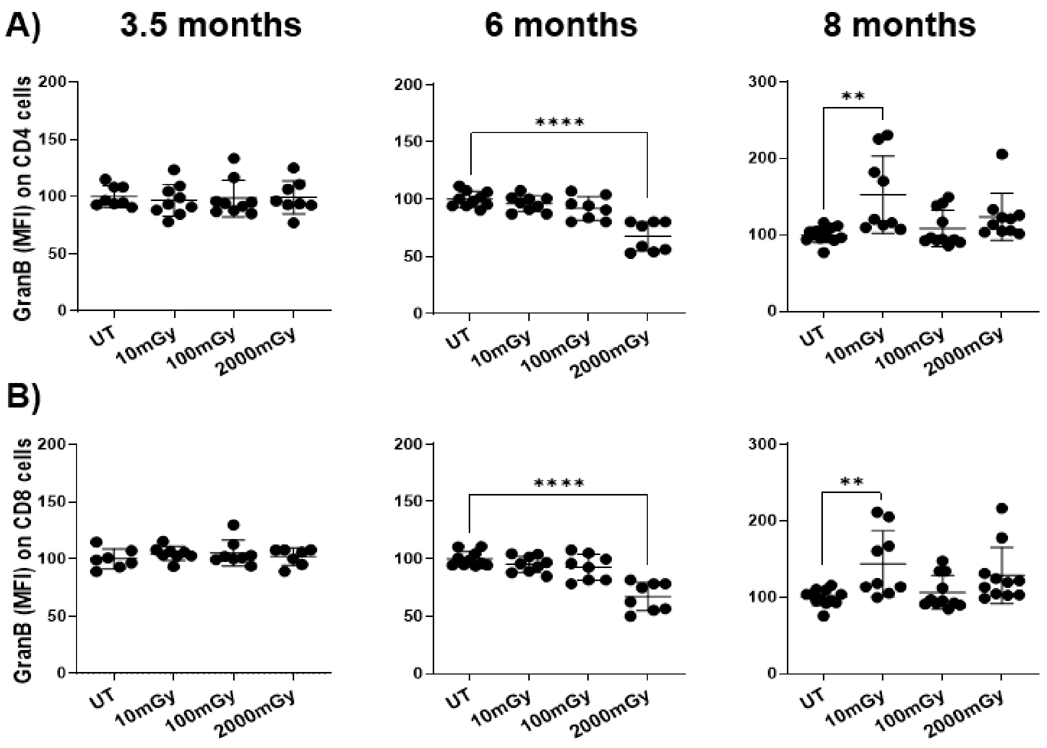

Supplementary Figure 4. Effect of LDR on T cell function.
Intracellular staining of splenic lymphocytes obtained from mice sacrificed at the indicated time of age after LDR exposure was performed to measure mean fluorescent intensity (MFI) of Granzyme B on A) TCRb+ CD4+ (CD4) cells and B) TCRb+ CD8+ (CD8) cells by flow cytometry. Y-axis represents the relative MFI of GranB on cells. Percent cells and MFI in untreated mice were set to 100%. Data represent mean + SD. ** $p < 0.01$, **** $p < 0.0001$).
